# Src Plays an Important Role in AGE-Induced Endothelial Cell Proliferation, Migration, and Tubulogenesis

**DOI:** 10.3389/fphys.2018.00765

**Published:** 2018-06-21

**Authors:** Peixin Li, Deshu Chen, Yun Cui, Weijin Zhang, Jie Weng, Lei Yu, Lixian Chen, Zhenfeng Chen, Haiying Su, Shengxiang Yu, Jie Wu, Qiaobing Huang, Xiaohua Guo

**Affiliations:** Key Laboratory for Shock and Microcirculation Research of Guangdong Province, Department of Pathophysiology, Southern Medical University, Guangzhou, China

**Keywords:** advanced glycation end products, Src, endothelial cells, ERK, angiogenesis

## Abstract

Advanced glycation end products (AGEs), produced by the non-enzymatic glycation of proteins and lipids under hyperglycemia or oxidative stress conditions, has been implicated to be pivotal in the development of diabetic vascular complications, including diabetic retinopathy. We previously demonstrated that Src kinase played a causative role in AGE-induced hyper-permeability and barrier dysfunction in human umbilical vein endothelial cells (HUVECs). While the increase of vascular permeability is the early event of angiogenesis, the effect of Src in AGE-induced angiogenesis and the mechanism has not been completely revealed. Here, we investigated the impact of Src on AGE-induced HUVECs proliferation, migration, and tubulogenesis. Inhibition of Src with inhibitor PP2 or siRNA decreased AGE-induced migration and tubulogenesis of HUVECs. The inactivation of Src with pcDNA3/flag-Src^K298M^ also restrained AGE-induced HUVECs proliferation, migration, and tube formation, while the activation of Src with pcDNA3/flag-Src^Y530F^ enhanced HUVECs angiogenesis alone and exacerbated AGE-induced angiogenesis. AGE-enhanced HUVECs angiogenesis *in vitro* was accompanied with the phosphorylation of ERK in HUVECs. The inhibition of ERK with its inhibitor PD98059 decreased AGE-induced HUVECs angiogenesis. Furthermore, the inhibition and silencing of Src suppressed the AGE-induced ERK activation. And the silencing of AGEs receptor (RAGE) inhibited the AGE-induced ERK activation and angiogenesis as well. In conclusions, this study demonstrated that Src plays a pivotal role in AGE-promoted HUVECs angiogenesis by phosphorylating ERK, and very likely through RAGE-Src-ERK pathway.

## Introduction

As one of the microvascular complications of diabetes, proliferative diabetic retinopathy (PDR) poses a threat to the vision of patients with diabetes (You et al., 2013). The pathological process mainly involves the apoptosis of pericytes, the disruption of blood retinal barrier, the increase of vascular permeability, the thickening of capillary basement membrane, and the pathological angiogenesis (Patz, 1982; Frank, 2004; Park et al., 2014). Angiogenesis is the process of forming new capillaries mainly involving the coordinated control of the permeability of microvessels, the migration, proliferation, polarity, and differentiation of endothelial cells, the deposition of basement membrane, and finally the formation and maturation of tubules (Herbert and Stainier, 2011). The occurrence of abnormal angiogenesis fuels the neovascularization of tumor and enhances tumor growth and progression to metastasis. Excessive angiogenesis is also a critical pathological process in the development of macular edema as well as PDR. To date, substantial success has been achieved by targeting angiogenesis in cancer and eye diseases.

Advanced glycation end products are a group of proteins or lipids produced by a non-enzymatic reaction between ketones or aldehydes with amino groups of proteins, lipids, and even nucleic acids (Takeuchi et al., 2010; Manigrasso et al., 2014). AGEs accumulated in the vessel wall may damage the structure and function of vascular cells and alter some functional properties of collagen laminin and vitronectin in extracellular matrix. For example, it was reported that the glycation of vitronectin by incubating with methylglyoxal (MGO), a glucose degradation product, inhibits VEGF-induced angiogenesis in HUVECs (Wang et al., 2015). Elevated levels of AGEs in the blood are proved to play a critical role in the development of PDR, in which angiogenesis presents as a major pathological manifestation (Vlassara et al., 1992; Yamagishi et al., 1997; Stitt et al., 2002; Kandarakis et al., 2014). By binding with receptor for AGEs (RAGE), AGEs elicit oxidative stress and inflammatory reactions subsequently (Sena et al., 2012). The accumulation of AGEs may impact the function of angiogenic activators such as basic fibroblast growth factor (bFGF), VEGF, and angiogenin-2 (Ang-2), etc. (Giardino et al., 1994; Okamoto et al., 2002). Therefore, better understanding of the effect and exact underlying mechanisms of AGEs on angiogenesis is urgently needed as it can provide us with a feasible and promising therapeutic approach for the vascular complications in T2D patients.

Src family kinases are the largest family of non-receptor tyrosine kinase with members of Src, Fyn, Blk, Yes, Lck, Lyn, Hck, Fgr, and Yrk, These various members of SFKs share similar structures covering a N-terminal 14-carbon myristoyl group, a particular segment, a SH2 domain, a SH3 domain, a protein–tyrosine kinase domain, and a C-terminal regulatory tail. The main phosphorylating and transforming activities of Src kinase are upregulated by Tyr419 phosphorylation and downregulated by Tyr530 phosphorylation. Src was activated when dephosphorylated at Y530 and auto-phosphorylated at Y419, and the latter can be used as markers for activated Src kinase. SFKs share four Src homology (SH) domains closely related to catalytic activity, interaction between proteins, and binding of cell membrane. They are characterized by signaling enzymes that regulate critical cellular processes such as proliferation, survival, migration, apoptosis, cell cycle control, and angiogenesis. One of well-known functions of phosphorylated SFKs is promoting cytoskeleton contraction. Especially, the contraction of endothelial cells will lead to the formation of intercellular gaps and increase of vascular permeability. Junctional complexes are also affected by SFKs through the phosphorylation of VE-cadherin, which leads to the disruption of cadherin–actin complex and subsequently endothelial hyper-permeability (Roura et al., 1999; Al Moustafa et al., 2002; Lambeng et al., 2005; Li et al., 2012). SFKs also impact vascular permeability by regulating extracellular matrix through focal adhesion complexes comprising integrins, FAK, paxillin and multiple adaptor proteins. Thus, SFKs affect not only endothelial cell shape but also cellular migration, as well as vascular permeability. In our previous studies, it has been proved that Src phosphorylated at Tyr 419 transduced the signal of AGEs through the ligation of RAGE to moesin, VE-cadherin, and FAK, resulting in the breakdown of endothelial barrier and vascular hyper-permeability (Zhang et al., 2015). The exudation of albumin from vascular space can be the early event of neovascularization.

It has been shown that the activation of Src acted as a node in signal transduction of several growth factor receptors (Eliceiri et al., 1999). The downstream signal transduction pathways of Src include PI3K, FAK, and mitogen-activated protein kinase (MAPK) (Golas et al., 2005; Jallal et al., 2007; Mayer and Krop, 2010; Delle Monache et al., 2014; Kumar et al., 2016). Known as the ERK pathway, the Ras/Raf/MEK/ERK signaling pathway plays a crucial role in neovascularization. Therefore, the aim of this study was to explore the role of SFK in AGE-mediated neovascularization and the possible effects of interaction between Src and ERK on this process.

## Materials and Methods

### Chemicals, Drugs, and Reagents

Human umbilical vein endothelial cells used in the experiments were purchased from Sciencell (San Diego, CA, United States). The coding sequences of oligonucleotide for Src, RAGE, and control siRNA were acquired from GenePharma (Shanghai, China). Here, are the siRNA-targeted sequences: control nonsense siRNA, AATTCTCCGAACGTGTCACGT; Src siRNA, GTTCGGAGGCTTCAACTCCT; RAGE siRNA, GGAATGGAAAGGAGACCA (Bjorge et al., 2011; Zhang et al., 2015). The mutants at Lys298 (kinase deficiency flag-Src-K298M, K298M) and at Tyr530 (constitutive active flag-Src-Y530F, Y530F) were synthesized by Genechem company (Shanghai, China). The full-length cDNA of Src was obtained by RT-PCR and was ligated into vector pcDNA3/FLAG to obtain the recombinant plasmid pcDNA3/FLAG-Src. The two mutants, pcDNA3.1/FLAG-Src^Y530F^ and pcDNA3.1/FLAG-Src^K298M^, were generated using primer forward of Y530F and K298M, respectively. The primers Y530F forward are 5′-ACGGGCCCTCTAGACTCGAGATGGGTAGCAACAAGAGCAAG-3′ and 5′-AGTCACTTAAGCTTGGTACCGAGAGGTTCTCCCCGGGCTGGT-3′. And primers K298M forward are 5′-ACGGGCCCTCTAGACTCGAGATGGGTAGCAACAAGAGCAAG-3′ and 5′-AGTCACTTAAGCTTGGTACCGAGAGGTTCTCCCCGGGCTGGAACTGGGGCTCGGTGGACG-3′. The mutations were identified using nucleotide sequencing (Zhang et al., 2015). Antibody against p-ERKThr202/Tyr204 (#4370, CST, Danvers, MA, United States), Src (#2108, CST, Danvers, MA, United States), p-SrcTyr416 (#2101, CST, Danvers, MA, United States), and RAGE (MAB-11451-100, R&D, Minneapolis, MN, United States) were purchased. The Isolectin B4 was obtained from Sigma (St. Louis, MO, United States). Src inhibitor PP2 (Cat# 529573) was acquired from Merck (Darmstadt, Germany) and ERK inhibitor PD98059 was purchased from Selleck, Inc. (Houston, TX, United States). The concentration of PP2 is 15 μmol/L and concentration of PD98059 used in this study is 20 μmol/L. Matrigel was purchased from Corning, Inc. (Corning, NY, United States). Secondary antibody for immunoblotting was manufactured by Sigma (St. Louis, MO, United States).

### Preparation of AGE-BSA

Advanced glycation end product-BSA was prepared in the light of protocol of Hou et al. (2001). AGEs were prepared by incubating BSA (150 mmol/L, pH 7.4) with D-glucose (250 mmol/L) in phosphate buffer at 37°C for 8 weeks. Then AGEs were disinfected with 0.22 μm filters. The AGE-modified protein concentration was measured through a BCA protein assay kit. Here, we used Pierce endotoxin removing gel to remove endotoxin and made sure the concentration of endotoxin was less than 500 EU/L, which was evaluated by ELISA kit (# MA1-83137, Thermo Fisher, Waltham, MA, United States).

### Cell Culture

Primary HUVECs were cultured in ECM added with FBS (5–10%), penicillin (100 units/mL) and streptomycin (100 μg/mL) at 37°C with 5% CO_2_. HUVECs were harvested when grew 80–90% confluence and starved of serum for 12 h before different treatments in this experiments. HUVECs at passages 4–6 were used in the experiments.

### Cell Viability Assay

The cell viability was measured by using cell counting kit-8 (CCK-8, Dojindo Molecular Technologies, Inc., Kumamoto, Japan). Cells were planted in 96-well culture plates and treated accordingly for different purposes. After pretreated with inhibitors, siRNA, or plasmids, cells were then stimulated with or without AGEs for 24 h. The media was then removed and CCK-8 at a concentration of 0.5 mg/mL was added. After 4 h, the absorbance at 450 nm was measured and the HUVECs proliferation capacity was assessed directly using optical density value (OD).

### Endothelial Cell Migration Assay

In scratch wound healing assay, 5 × 10^5^/mL HUVECs were seeded in 6-well plates to grow to confluent monolayer. A 10 μL pipette tip was used to scratch the cell monolayers, leaving a 400–600 μm gap. The initial area in different groups were kept as similar as possible. After pretreated with inhibitors, siRNA, or plasmids, cells were cultured with or without AGEs, respectively for 24 h. Then wound healing pictures were obtained by using a phase contrast microscope, and Image J was used to analyze the images. The HUVECs migration area was calculated as: [open image area at 24 h/initial open image area] × 100%.

Human umbilical vein endothelial cells migration was evaluated by using 8 μm pore-sized filter transwell (Corning, NY, United States). 100 μL HUVECs suspension at 2–3 × 10^5^/ml was seeded in the upper chamber. ECM was used in the lower chamber as a chemoattractant. After incubation at 37°C for 24 h with different stimulation, cells in the upper chamber were erased and cells moved to the lower surface of the filters were fixed using 4% polyoxymethylene, dyed with crystal violet and photographed with a microscope. The numbers of cells migrated to the lower surface of the filters were calculated.

### Capillary Tube Formation Assay

Fifty μL Matrigel (Corning, Inc., Corning, NY, United States) was added to a 96-well plate and the gel was allowed to concrete at 37°C for 1 h. HUVECs were harvested and re-suspended to 2 × 10^5^/ml in ECM. 100 μL cell suspension was plated in 96-well plate precoated with Matrigel. HUVECs were treated with inhibitors or transfected with siRNA or plasmids respectively, followed by 24 h of incubation with or without AGEs. The plates were photographed under a phase contrast microscope. The total tube length, the branch points and the vessel area was measured using Image J.

### Western Blot

Total cellular extracts were prepared with lysis buffer (20 mM Tris-HCl pH 7.4, 2.5 mM EDTA, 1% Triton X-100, 0.1% SDS, 100 mmol/L NaCl, 1% deoxycholic acid, 1 mM Na_3_VO_4_,10 mM NaF) with protease and phosphatase inhibitors. The cell lysates were collected after 12,000 rpm centrifugation for 10 min at 4°C, and heated at 100°C for 5 min. Proteins were loaded and subjected to SDS-PAGE subsequently transferred to polyvinylidene fluoride (PVDF) membranes. The membrane was blocked with 5% bovine serum albumin in TBS containing 0.5% Tween 20 (TBS-T) for 1 h and incubated overnight at 4°C with a 1:1000 dilution of primary antibody against ERK, p-ERK, Src, p-Src, and RAGE. After washing three times with TBS-T for 5 to 10 min each time, the blots were incubated with secondary antibody for 1 h at 25°C. After triple washes for 10 min each time, visualization of protein bands was performed using electro-chemiluminescence (ECL). Densitometric analysis was operated in Kodak IS2000R imaging station. GAPDH was used as an internal control.

### Transfection of HUVECs With siRNA and Plasmid

Human umbilical vein endothelial cells were transfected with Src, RAGE or control nonsense siRNA (20 nM) using siRNA-MateTM according to the protocol provided by GenePharma (Shanghai, China) after the cells were cultured in ECM and grew to 30–50% confluence. 48 h after transfection, total protein was prepared and subjected to immunoblotting with anti-Src or RAGE antibody to verify siRNA-mediated knockdown in Src and RAGE expression. HUVECs were transfected with plasmids with LipoFilter^TM^ Liposomal Transfection Reagent (Genechem, Shanghai, China) when cells grew to 70–90% confluence. To be brief, the DNA mixture with 250 μl DMEM plus 0.4 μg DNA and 12 μl LipoFilter^TM^ was added to HUVECs in 6-well plates and incubated for 48 h followed by treatments with or without AGEs.

### Mouse Aortic Ring Assay

Mouse thoracic aortas were prepared according to the protocol of Baker et al. (2012). Thoracic aortas were removed from 8 to 12 weeks old male C57 mice and moved to a culture dish with cold serum-free Dulbecco’s minimal essential medium (D-MEM, Gibco, Invitrogen). The tissue was carefully removed with micro dissecting tweezers and scissors, minding not to damage the aortic wall. 1 to 2 mm-long aortic slices (about 20 per one aorta) were sectioned and incubated in Opti media containing 2.5% fetal bovine serum overnight. The aorta rings were then embedded on collagen IV coated 96-well plates. Opti medium containing PP2 or PD98059 was added and the plates were incubated at 37°C, 5% carbon dioxide for 90 or 30 min. Fresh medium with or without AGEs was reintroduced into the plates and changed every other day for another 6 days. The rings were examined by a Zeiss microscope. We estimated the amount of the capillary from the aortic rings. The rings were incubated with 0.1 mg/mL isolectin B4 from Sigma (St. Louis, MO, United States) to stain endothelial cells. The images were obtained under an epifluorescence microscopes (Zeiss).

### Statistical Analysis

GraphPad Prism version 7.0 and SPSS 22.0 software was used to analyzed the data. All data were expressed as means ± SD of at least three independent experiments. Statistical comparisons were performed using one-way ANOVA and *P* < 0.05 was considered significant.

## Results

### Role of Src in AGE-Induced Angiogenesis

**FIGURE 1:**
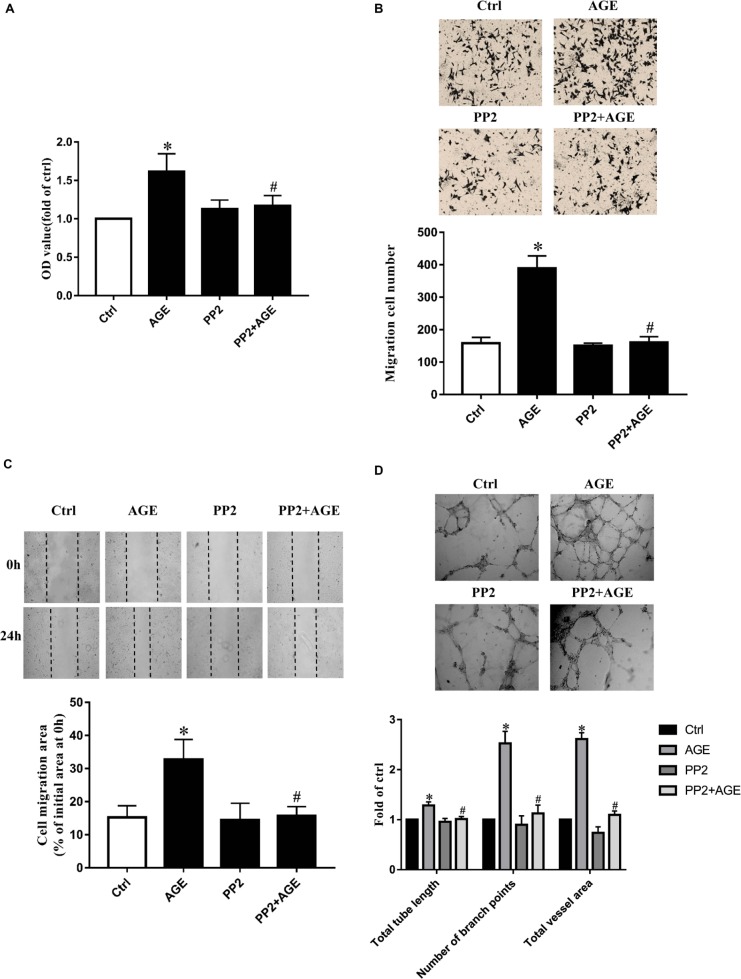
Src inhibitor PP2 attenuated AGE-enhanced HUVECs proliferation, migration, and tube formation. HUVECs were incubated with 15 μmol/L PP2 for 90 min and then exposed to 100 μg/mL AGEs for 24 h. CCK-8 was used to evaluate the proliferation of HUVECs **(A)**. Scratch wound healing **(B)** and transwell migration assay **(C)** were used to measure the migration of HUVECs. The tube length, numbers of branch points, and vessel area were observed in Matrigel medium **(D)**. *n* ≥ 4 independent experiments. ^∗^*P* < 0.05 versus control, ^#^*P* < 0.05 versus AGEs.

Human umbilical vein endothelial cells were incubated with 100 μg/mL AGEs for 24 h to explore the effects of AGEs on endothelial proliferation, migration, and tube formation. The culture medium was used as a blank control. Compared to the control group, HUVECs proliferation was significantly enhanced by the treatment of AGEs (*P* < 0.05) (**Figure 1A**). The application of AGEs also promoted the wound healing and transwell migration in HUVECs (**Figures 1B,C**). HUVECs tube formation was obviously increased by AGEs treatment as well (*P* < 0.05) (**Figure 1D**). Based on these results and our previous study (Wang et al., 2016), we believed that AGEs have the potential to induce angiogenesis. Previously study in our laboratory has confirmed AGEs can activate Src kinase (Zhang et al., 2015). Here we speculated that Src would also participate in the signal transduction of AGE-induced angiogenesis. HUVECs were then pre-incubated with PP2, a Src specific inhibitor, for 90 min before AGEs treatment to block Src activation, the culture medium was used as a blank control. Compared to AGE-treated alone, the application of PP2 significantly attenuated AGE-induced proliferation and migration of HUVECs (*P* < 0.05) (**Figures 1A–C**). The effect of AGEs in HUVECs tube formation was also abolished by PP2 (*P* < 0.05) (**Figure 1D**). These results indicated that Src was involved in AGE-induced angiogenesis.

**FIGURE 2:**
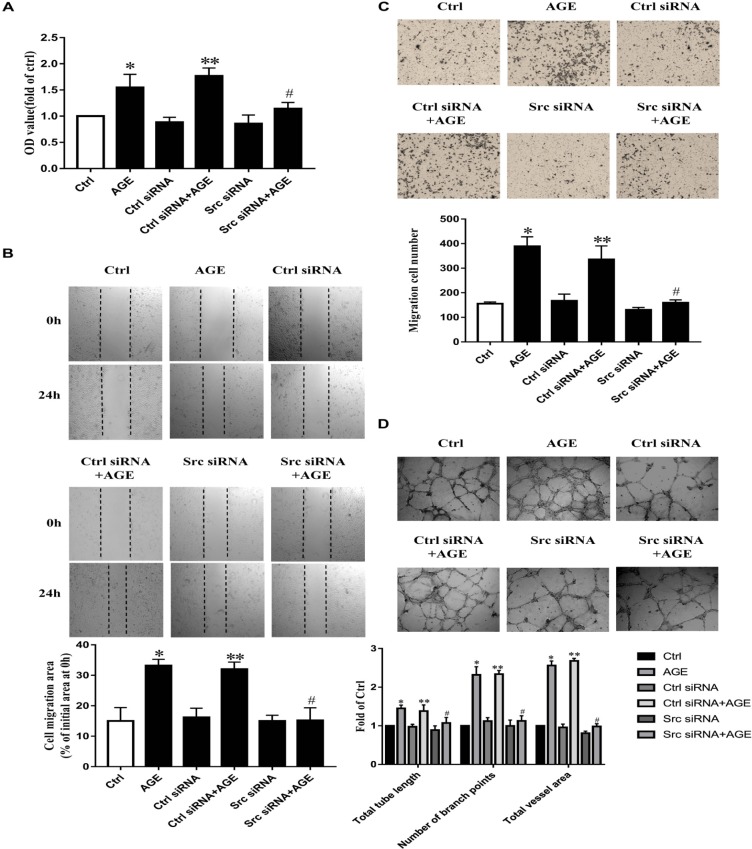
Src siRNA weakened AGE-induced endothelial angiogenesis. HUVECs were transfected with Src siRNA or control siRNA for 48 h and then treated with 100 μg/mL AGEs for 24 h. CCK-8 was used to evaluate the proliferation of HUVECs **(A)**. Scratch wound healing **(B)** and transwell migration assay **(C)** were used to measure the migration of HUVECs. The tube length, numbers of branch points, and vessel area were observed in Matrigel medium **(D)**. *n* ≥ 4 independent experiments. ^∗^*P* < 0.05 versus control, ^#^*P* < 0.05 versus AGEs, ^∗∗^*P* < 0.05 versus Control siRNA.

To further validate the role of Src in AGE-induced angiogenesis, we transfected HUVECs with Src siRNA, the culture medium was used as a blank control and the control siRNA was used as siRNA basal control, respectively. The efficiency of suppressed expression of siRNA on Src was confirmed (Supplementary Figure 1A). AGEs could enhance the proliferation (**Figure 2A**) (*P* < 0.05), migration (*P* < 0.05) (**Figures 2B,C**), and tube formation (*P* < 0.05) (**Figure 2D**) in HUVECs transfected with control siRNA, however, failed to consist these effects on HUVECs transfected with Src siRNA. These results showed that Src was necessary in AGE-induced endothelial angiogenesis.

### The Involvement of Tyrosine Kinase Activity of Src in Manipulation of AGE-Induced Angiogenesis

To further confirm whether the tyrosine kinase activity of Src was associated with AGE-induced angiogenesis, we transfected HUVECs with plasmids with a kinase-deficient mutant at Lys298 (K298M) or an active mutant at Tyr530 (Y530F). The mock plasmid was used as a mutant control. The effects of relevant plasmids on Src phosphorylation were confirmed (Supplementary Figure 1C).

**FIGURE 3:**
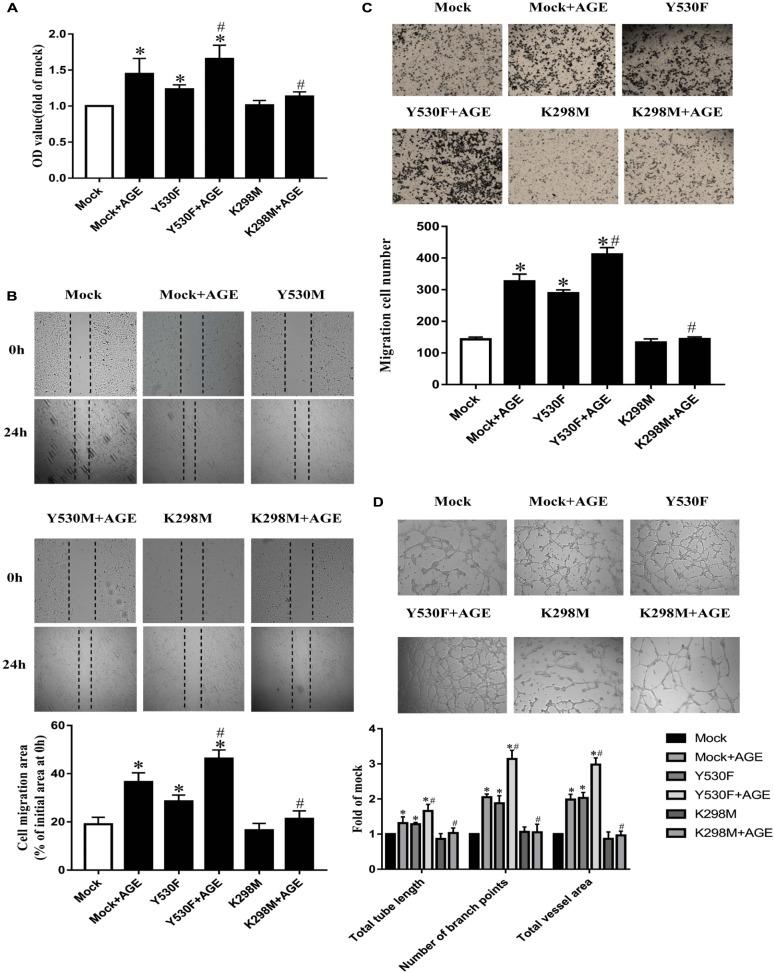
Tyrosine kinase activity of Src was associated with AGE-induced endothelial angiogenesis. HUVECs were transfected with a kinase-deficient mutant at Lys298 (K298M) and an active mutant at Tyr530 (Y530F), also with a Mock as plasmid control. 48 h after transfection, HUVECs were incubated with 100 μg/mL AGEs for 24 h. CCK-8 was used to evaluate the proliferation of HUVECs **(A)**. Scratch wound healing **(B)** and transwell migration assay **(C)** were used to measure the migration of HUVECs. The tube length, numbers of branch points, and vessel area were observed in Matrigel medium **(D)**. *n* ≥ 4 independent experiments. ^∗^*P* < 0.05 versus Mock, ^#^*P* < 0.05 versus Mock + AGEs.

The proliferation, migration, and tube formation were promoted in HUVEC-transfected with pcDNA3/flag-Src^Y530F^ alone (*P* < 0.05) (**Figures 3A–D**). And the effects of pcDNA3/flag-Src^Y530F^ on HUVECs were further exacerbated when AGEs was added (*P* < 0.05) (**Figures 3A–D**). On the opposite, transfected with mock plasmid did not change AGE-induced angiogenesis. While transfected with pcDNA3/flag-Src^K298M^, AGE-induced proliferation, migration, and tube formation were significantly attenuated (*P* < 0.05) (**Figures 3A–D**). These results demonstrated that tyrosine kinase activity of Src was necessary in manipulating AGE-induced angiogenesis.

All the above results indicated that the activation of Src played a critical role in the signaling of AGE-induced angiogenesis.

### Src Activation Modulated AGE-Induced Angiogenesis by Enhancing ERK Phosphorylation

**FIGURE 4:**
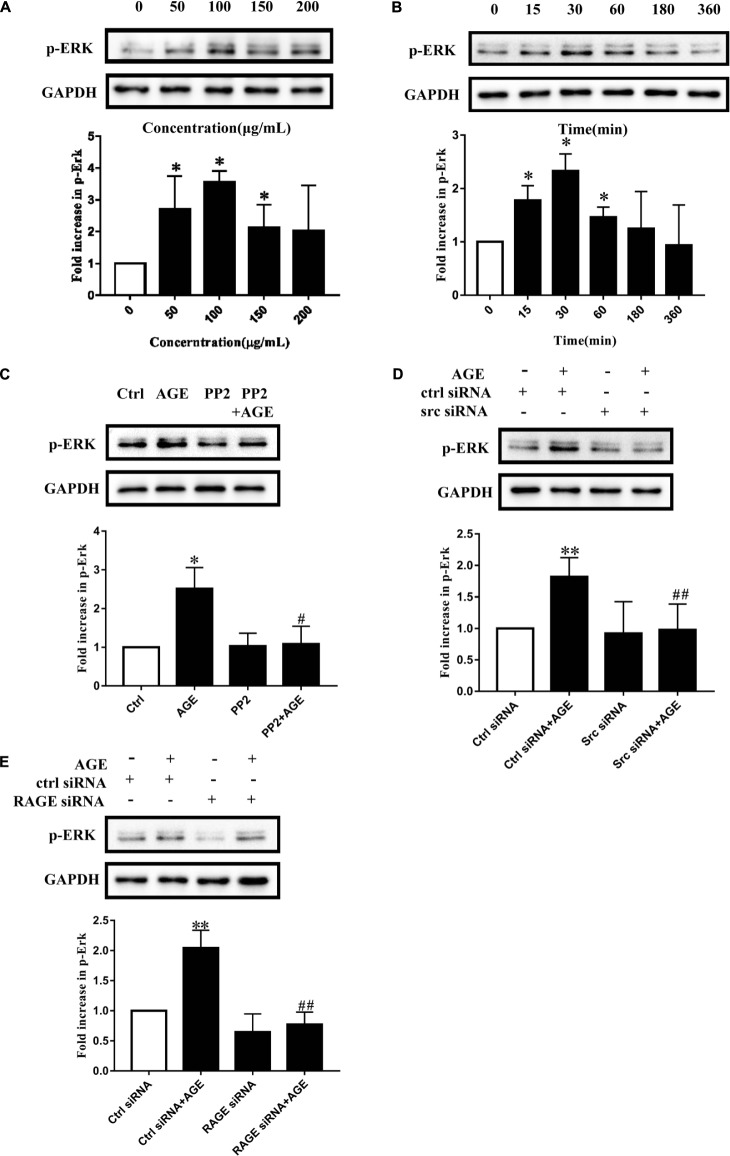
Advanced glycation end products induced ERK phosphorylation required Src and RAGE. HUVECs were treated with 100 μg/mL AGEs for 15, 30, 60, 180, 360 min **(A)** and the phosphorylation level of ERK was detected by Western Blot. To investigate the effect of AGEs concentration on ERK phosphorylation, HUVECs were incubated with 50, 100, 150, 200 μg/mL AGEs for 30 min **(B)**. To study the role of Src in AGE-induced ERK phosphorylation, HUVECs were pretreated with 15 μmol/L PP2 for 90 min and then incubated with 100 μg/mL AGEs for 30 min **(C)**. HUVECs were transfected with Src siRNA or control siRNA for 48 h and cultured with 100 μg/mL AGEs for 30 min to further investigate the effect of Src **(D)**. Also, we transfected HUVECs with RAGE siRNA or control siRNA for 48 h and then incubated with 100 μg/mL AGEs for 30 min to further investigate the effect of RAGE **(E)**. *n* ≥ 4 independent experiments. ^∗^*P* < 0.05 versus Control, ^#^*P* < 0.05 versus AGEs, ^∗∗^*P* < 0.05 versus Control siRNA, ^##^*P* < 0.05 versus Control siRNA + AGEs.

Extracellular signal-regulated kinase is a well-known proliferative MAPK and is also implicated as a conventional downstream signal of Src. We speculated that Src activation would modulate AGE-induced angiogenesis by affecting ERK phosphorylation in endothelial cells. To find out the concentration effect of AGEs on ERK phosphorylation, we cultured HUVECs with different dose of AGEs and the ERK phosphorylation level was detected by western blot. The ERK phosphorylation reached a peak when HUVECs were exposed to 100 μg/mL AGEs (*P* < 0.05) (**Figure 4A**). HUVECs were also cultured with 100 μg/mL AGEs for various duration, and ERK phosphorylation was enhanced at 15 min, reached a peak at 30 min and then returned to baseline level at 6 h (*P* < 0.05) (**Figure 4B**). Pretreatment of HUVECs with PP2 attenuated AGE-induced ERK phosphorylation (*P* < 0.05) (**Figure 4C**). Furthermore, pretransfected with Src siRNA abolished AGE-induced ERK phosphorylation too (*P* < 0.05) (**Figure 4D**). In order to find out the effect of RAGE on AGE-induced ERK phosphorylation, we transfected HUVECs with RAGE siRNA (Supplementary Figure 1B). And the results showed that AGE-induced ERK phosphorylation could be attenuated by silencing RAGE (*P* < 0.05) (**Figure 4E**). These results indicated that AGEs could induce ERK phosphorylation through Src pathway and it’s required for AGE–RAGE binding.

### AGEs Exerted the Effects on Src Activation and Pro-angiogenic Through RAGE

**FIGURE 5:**
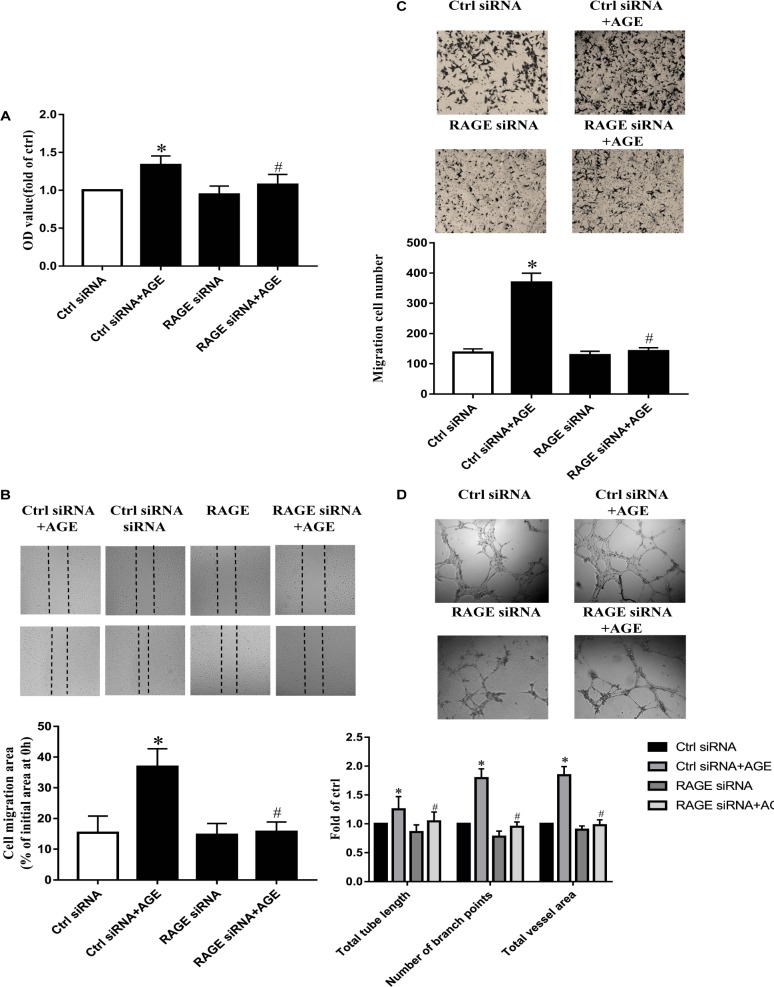
The role of ERK in AGE-induced angiogenesis. HUVECs were pretreated with 20 μmol/L PD98059 for 30 min and then incubated with 100 μg/mL AGEs for 24 h. CCK-8 was used to evaluate the proliferation of HUVECs **(A)**, scratch wound healing **(B)** and transwell migration assay **(C)** were used to measure the migration of HUVECs. The tube length, numbers of branch points, and vessel area were observed in Matrigel medium **(D)**. *n* ≥ 4 independent experiments. ^∗^*P* < 0.05 versus control, #*P* < 0.05 versus AGEs.

Our previous study has proved that AGE-induced Src activation relied on the interaction of AGEs with RAGE (Zhang et al., 2015). Here again, we confirmed that AGE-induced angiogenesis in HUVECs needed the participation of RAGE. The suppressed expression of RAGE by siRNA abolished the proliferation, migration, and tube formation in HUVECs treated with AGEs, while control siRNA did not alter the effects of AGEs on HUVECs (*P* < 0.05) (**Figures 5A–D**). These results indicated that AGEs might exert its pro-angiogenic effects through RAGE, and then Src.

### Role of ERK Phosphorylation in AGE-Induced Angiogenesis

**FIGURE 6:**
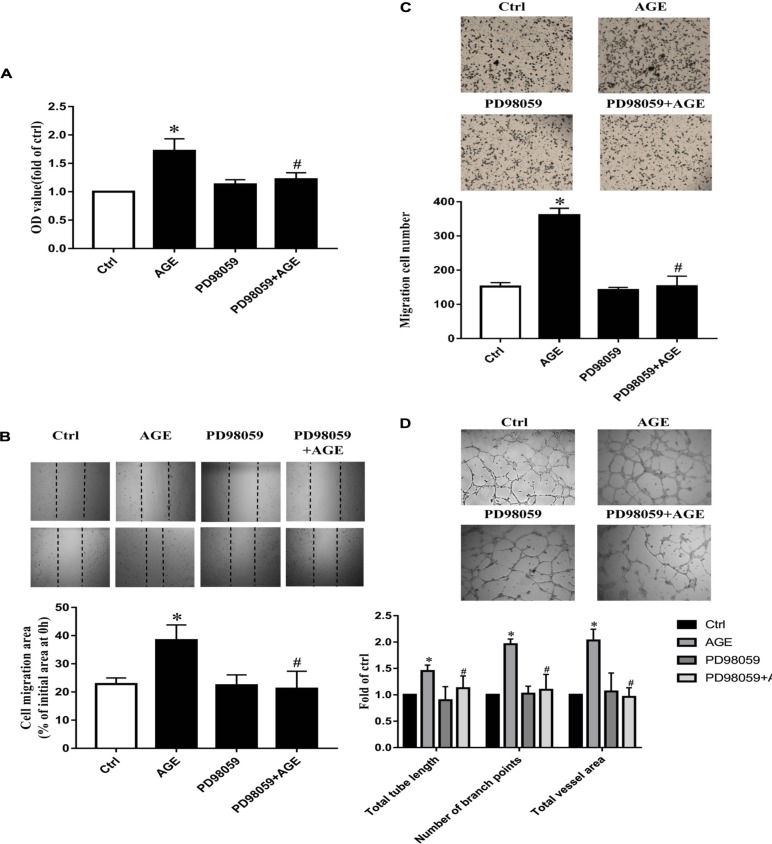
The role of RAGE in AGE-induced angiogenesis. HUVECs were pre-transfected with RAGE siRNA or control siRNA for 48 h and then cultured with 100 μg/mL AGEs for 24 h. CCK-8 was used to evaluate the proliferation of HUVECs **(A)**, scratch wound healing **(B)**, and transwell migration assay **(C)** were used to measure the migration of HUVECs. The tube formation was observed in Matrigel medium **(D)**. *n* ≥ 4 independent experiments. ^∗^*P* < 0.05 versus control, ^#^*P* < 0.05 versus AGEs.

To explore the role of ERK in AGE-induced angiogenesis, we used PD98059, a specific ERK inhibitor, to inactivate ERK. By pretreating HUVECs with 20 μmol/L PD98059 30 min before AGEs, the proliferation, and migration were all attenuated significantly compared to AGE-treated group (*P* < 0.05) (**Figures 6A–C**). PD980589 also abolished AGE-induced tube formation (*P* < 0.05) (**Figure 6D**). Combining with the effect of Src in AGE-induced EKR phosphorylation, the results indicated that Src activation modulated AGE-induced angiogenesis by enhancing ERK phosphorylation.

### Inhibition of Src and ERK Activation Attenuated AGE-Induced Angiogenesis in Mouse Aortic Ring

**FIGURE 7:**
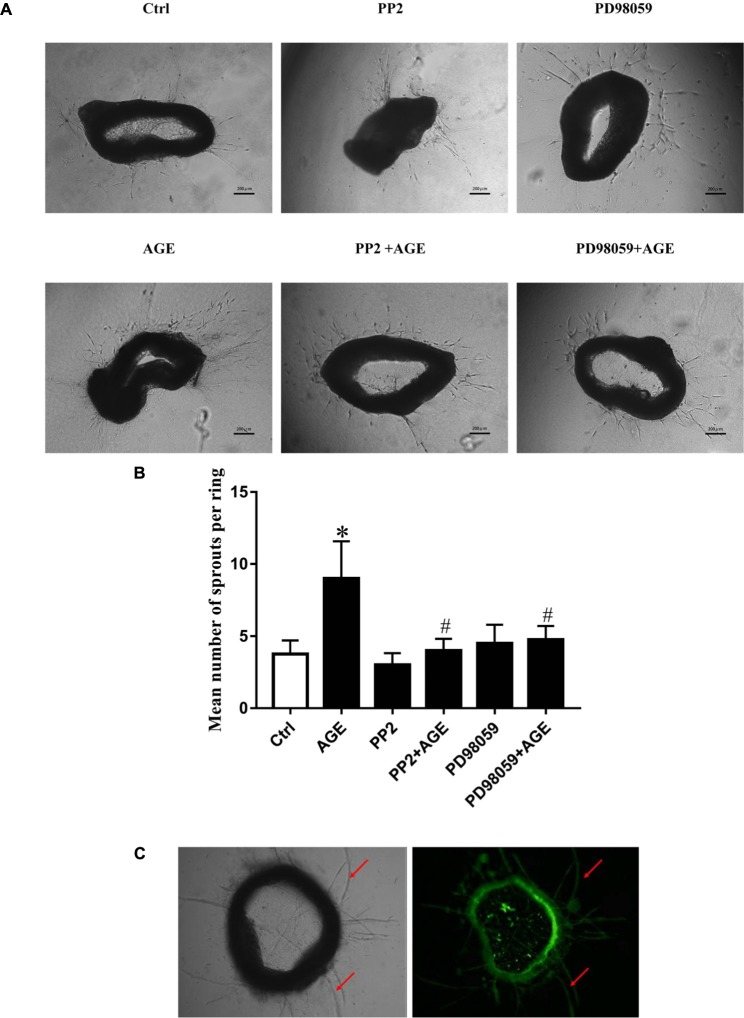
The Role of Src/ERK pathway in AGE-induced aortic ring sprouting. The aortic rings were pretreated with 15 μmol/L PP2 for 90 min or 20 μmol/L PD98059 for 30 min respectively and then incubated with 100 μg/mL AGEs for 6 days. Fresh medium with or without AGEs was reintroduced every 2 days. An estimation of the capillary was performed by counting the branches from the aortic explants. Graph **B** displayed the statistical results of angiogenesis in mouse aortic ring. **(A,B)**. *n* = 3 independent experiments. ^∗^*P* < 0.05 versus control, #*P* < 0.05 versus AGEs. Scale bar: 200 μm. The aortic rings were incubated with isolectin B4 (green) for 1 h to stain endothelial cells for the indication of sprouts of neovessels (red arrows) **(C)**.

To further verify the effects of Src/ERK signal pathway on AGE-induced angiogenesis, mouse aortic ring angiogenesis assay was applied in this study. The results showed that AGEs treatment significantly increased the number of sprouting vessels, while the application of Src inhibitor PP2 or ERK inhibitor PD98059 could abolished the increase of sprouting vessel numbers in AGE-treated aortic rings (**Figure 7**).

## Discussion

Increased vascular endothelial monolayer permeability provides suitable environment for sprouting of a new capillary, follows by the activation, proliferation and migration of endothelial cells, and finally leads to angiogenesis. Divergent angiogenic responses occur in different organs in diabetic state under the influence of different angiogenic factors, such as VEGF, FGF and EGF and their respective receptors VEGFR, FGFR, and EGFR.

The increase of retinal neovascularization is a major cause of diabetic retinopathy. It has been elucidated that the accumulation of serum AGEs in diabetes increases the seriousness of microvascular complications. And it’s reported that AGEs accumulated in a hyperglycemic condition can affect angiogenesis depending on the microenvironment of the cells (Devi and Sudhakaran, 2011). In our research, compared with the control group, AGEs enhanced the proliferation, scratch wound healing process, transwell migration, tube formation in HUVECs, as well as angiogenesis in mouse aortic ring, manifesting the critical role of AGEs in angiogenesis.

It is now well-established that SFKs play an important role in cell cycle regulation, adhesion, migration, proliferation, and differentiation in different cells and tissues (Thomas and Brugge, 1997). As for angiogenesis, the effects of SFKs have been long demonstrated. It is proved that activated Src participated in signaling pathways giving rise to stimulation of endothelial cell survival and angiogenesis. Moreover, it has been found that Src kinase inhibitor PP2 inhibited hypoxia inducible factor 1α (HIF-1α) and STAT3 expression (Jiang et al., 1997; Gray et al., 2005). It indicates that Src-dependent signaling cascades are closely associated with angiogenesis. GLP-1 has a pro-angiogenic action on HUVECs and Src signaling pathways plays a key role as well (Aronis et al., 2013). S13, another specific Src kinase inhibitor, reduced angiogenic potential of human endothelial cells *in vitro* and *in vivo* (Delle Monache et al., 2014). And Src also plays a role in interleukin 18 (IL-18) induced angiogenesis (Amin et al., 2010). In general, Src kinases exert an irreplaceable influence on both tumor-induced neovascularization and inflammation-mediated angiogenesis. Our previous study demonstrated that Src phosphorylated at Tyr 419 acted like a signal node and gave rise to endothelial hyperpermeability upon stimulation of AGEs. Here, we demonstrated that Src can also play an irreplaceable role in angiogenesis induced by AGEs. Down-regulation of Src expression with siRNA or PP2 illustrated that inactivation of Src decreased AGE-induced HUVECs proliferation, migration, and tube formation. We also observed that those effects were attenuated by pcDNA3/flag-Src^K298M^, while increased by pcDNA3/flag-Src^Y530F^ comparing to mock group. These data ulteriorly proved that Src, especially when phosphorylated at Tyr 419, mediated AGE-induced angiogenesis.

Receptor for advanced glycation end product is a 35 kDa transmembrane receptor belonging to the immunoglobulin gene superfamily. It can be atypically up-regulated in diabetes complications, cardiovascular diseases, ischemic injury, cancer, and Alzheimer’s disease. Knockdown of RAGE expression could inhibit colorectal cancer angiogenesis *in vitro* and *in vivo* to some extent (Liang et al., 2011). Moreover, in our previous study, AGEs were affiliated with RAGE to activate Src. In this study, RAGE was proved to be required for ERK phosphorylation too (**Figure 4E**). And RAGE siRNA could prevent AGE-induced endothelial proliferation, migration, and tube formation (**Figure 6**). In general, it implied that RAGE was involved in Src mediated endothelial angiogenesis induced by AGEs.

Mitogen-activated protein kinase is a major signaling system involved in a variety of fundamental cellular processes such as proliferation, differentiation, motility, stress response, apoptosis, and survival. We verified that activated ERK in endothelial cells culminated at 30 min when exposed to AGEs at the concentration of 100 μg/mL (**Figure 4B**). VEGF and hypoxic condition can boost RF/6A endothelial cell proliferation and tube formation, while ERK inhibitor PD98059 can inhibit those effects (Jin et al., 2013). The endothelial cell proliferation and survival in tumor neovascularization are also dependent on the activation of ERK pathway in endothelial cells. It indicates that Src/ERK signal pathway plays a role in angiogenesis during the development of liver cirrhosis (Ding et al., 2015). So to further test the possible role of ERK in AGE-induced angiogenesis, we investigated whether Src kinase mediated cell signaling from AGE-RAGE binding to ERK phosphorylation and the angiogenic effect of ERK. The results showed that inhibition of endothelial cell ERK activation by the inhibitor PD98059 attenuated AGE-induced angiogenesis (**Figure 5**). And the phosphorylation of ERK was significantly abolished when Src kinase was silenced or inactivated (**Figures 4C,D**). Hence, we inferred that ERK was probably activated followed by the activation of Src, and participated in AGE-triggered angiogenesis.

## Conclusion

Our study indicated that AGEs could exert an angiogenic effect through the RAGE/Src/ERK signaling pathway. The findings suggested that Src might be an appropriate target for the prevention and treatment of AGEs associated microvasculopathy. Also, the ERK and cross-talking pathway could be a selective daughter node and reinforce the therapy. Since not only endothelial cells participate in the vessel regeneration, more research is needed on addressing the AGE-induced angiogenesis.

## Ethics Statement

Ethics approval and consent to participate. All animal experiments were approved by the Animal Care Committee of the Southern Medical University of China and strictly followed the Guide for the Care and Use of Laboratory Animals of the National Institutes of Health.

## Author Contributions

QH and XG conceived and arranged the collaboration, initiated the manuscript, edited and compiled the final version for submission. PL, DC, and LC carried out most of the experimental work. PL, WZ, and DC wrote the manuscript. YC, WZ, JWe, LY, ZC, HS, SY, and JWu helped in study design. All authors analyzed data, reviewed and approved the final manuscript.

## Conflict of Interest Statement

The authors declare that the research was conducted in the absence of any commercial or financial relationships that could be construed as a potential conflict of interest.

## Abbreviations

AGEs: advanced glycation end products
ECM: endothelial cell medium
EGF: epidermal growth factor
ERK: extracellular signal-regulated kinase
FAK: focal adhesion kinase
FGF: fibroblast growth factor
GLP-1: glucagon-like peptide 1
HUVECs: human umbilical vein endothelial cells
PI3K: phosphatidylinositol-3- kinase
RAGEs: receptor for advanced glycation end products
SFK: Src family kinases
VE-cadherin: vascular endothelial cadherin
VEGF: vascular endothelial growth factor
