# Supplementary material for: Src Plays an Important Role in AGE-Induced Endothelial Cell Proliferation, Migration, and Tubulogenesis
Source: Front Physiol. 2018 Jun 21;9:765. doi: 10.3389/fphys.2018.00765 (PMC6021521; doi:10.3389/fphys.2018.00765)
Supplement: Supplementary file 1 [file Data_Sheet_1.docx]

Supplementary Material

Src plays an important role in AGE-induced endothelial cell proliferation, migration and tubulogenesis

Peixin Li*, Deshu Chen*, Yun Cui, Weijin Zhang, Jie Weng, Lei Yu, Lixian, Chen Zhenfeng Chen, Haiying Su, Shengxiang Yu, Jie Wu, Qiaobing Huang, Xiaohua Guo

*** Correspondence:** Qiaobing Huang : [bing@smu.edu.cn](mailto:bing@smu.edu.cn)

Xiaohua Guo: [lanblue@smu.edu.cn](mailto:lanblue@smu.edu.cn)

**Supplementary Figure 1.** **The effects of Src siRNA, RAGE siRNA, pcDNA3/flag-Src^Y530F^ and pcDNA3/flag-Src^K298M^**

HUVECs were transfected with Src siRNA or control siRNA for 48h and the level of Src was detected (A). Also, to investigate the efficiency of RAGE siRNA, we transfected HUVECs with RAGE siRNA or control siRNA and detect the level of RAGE (B). The effects of pcDNA3/flag-Src^Y530F^ and pcDNA3/flag-Src^K298M^ on expression of Src were detected by western blotting as well (C). n ≥ 3 independent experiment. *P<0.05 versus control, ^#^P<0.05 versus mock.
