# Supplementary figures and images for: Src Plays an Important Role in AGE-Induced Endothelial Cell Proliferation, Migration, and Tubulogenesis
Source: Front Physiol. 2018 Jun 21;9:765. doi: 10.3389/fphys.2018.00765 (PMC6021521; doi:10.3389/fphys.2018.00765)

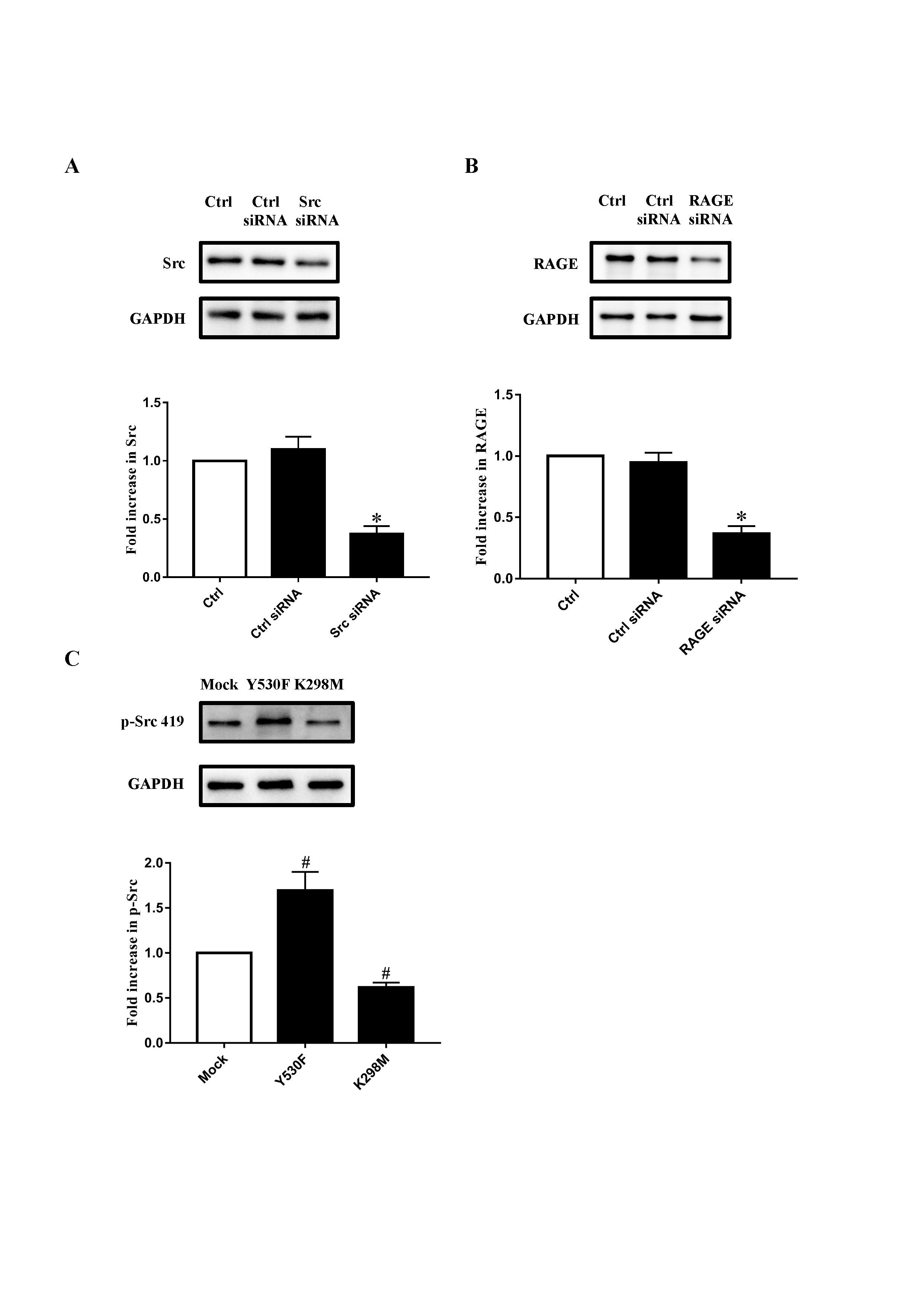

Supplement: Supplementary file 2 [file Image_1.jpg]
